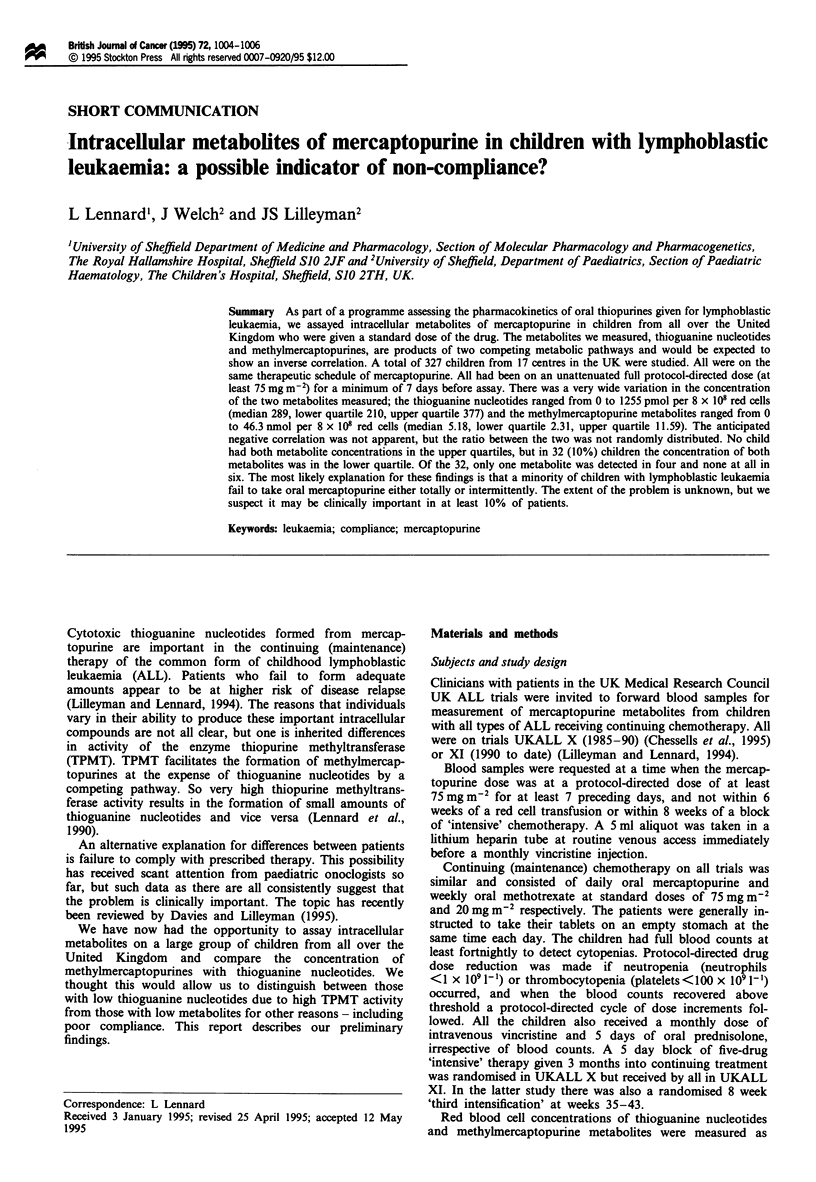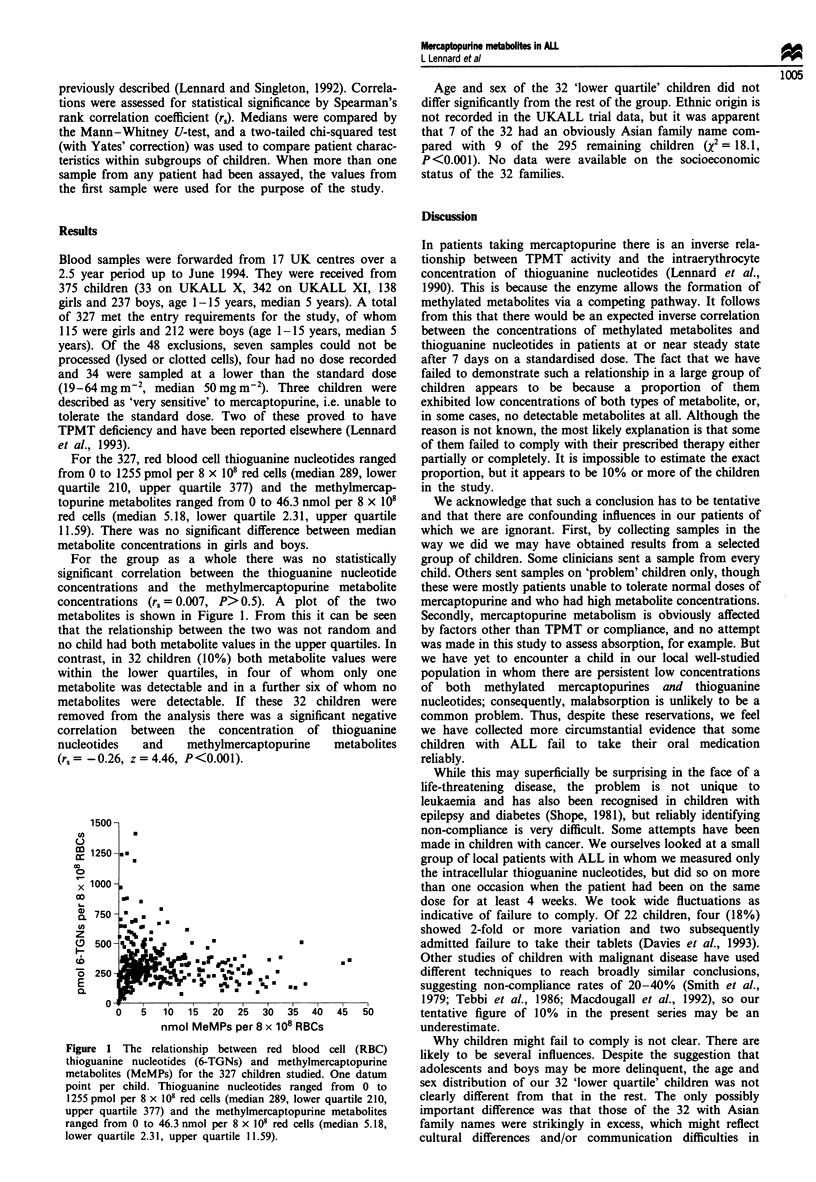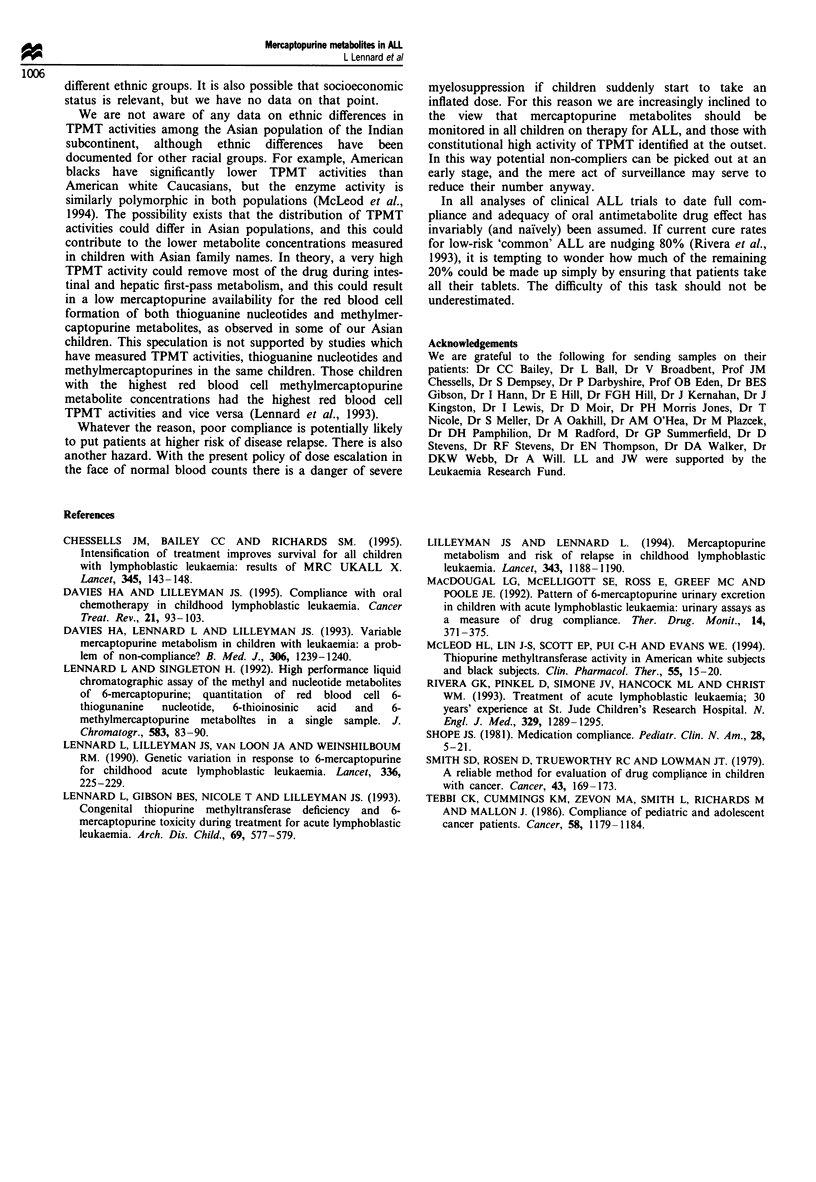# Intracellular metabolites of mercaptopurine in children with lymphoblastic leukaemia: a possible indicator of non-compliance?

**DOI:** 10.1038/bjc.1995.450

**Published:** 1995-10

**Authors:** L. Lennard, J. Welch, J. S. Lilleyman

**Affiliations:** University of Sheffield Department of Medicine and Pharmacology, Royal Hallamshire Hospital, UK.

## Abstract

As part of a programme assessing the pharmacokinetics of oral thiopurines given for lymphoblastic leukaemia, we assayed intracellular metabolites of mercaptopurine in children from all over the United Kingdom who were given a standard dose of the drug. The metabolites we measured, thioguanine nucleotides and methylmercaptopurines, are products of two competing metabolic pathways and would be expected to show an inverse correlation. A total of 327 children from 17 centres in the UK were studied. All were on the same therapeutic schedule of mercaptopurine. All had been on an unattenuated full protocol-directed dose (at least 75 mg m-2) for a minimum of 7 days before assay. There was a very wide variation in the concentration of the two metabolites measured; the thioguanine nucleotides ranged from 0 to 1255 pmol per 8 x 10(8) red cells (median 289, lower quartile 210, upper quartile 377) and the methylmercaptopurine metabolites ranged from 0 to 46.3 nmol per 8 x 10(8) red cells (median 5.18, lower quartile 2.31, upper quartile 11.59). The anticipated negative correlation was not apparent, but the ratio between the two was not randomly distributed. No child had both metabolite concentrations in the upper quartiles, but in 32 (10%) children the concentration of both metabolites was in the lower quartile. Of the 32, only one metabolite was detected in four and none at all in six. The most likely explanation for these findings is that a minority of children with lymphoblastic leukaemia fail to take oral mercaptopurine either totally or intermittently. The extent of the problem is unknown, but we suspect it may be clinically important in at least 10% of patients.


British Journal of Cancer (1995) 72, 1004–1006
© 1995 Stockton Press All rights reserved 0007–0920/95 $12.00

SHORT COMMUNICATION

### Intracellular metabolites of mercaptopurine in children with lymphoblastic leukaemia: a possible indicator of non-compliance?

L Lennard[1], J Welch[2] and JS Lilleyman[2]

[1]*University of Sheffield Department of Medicine and Pharmacology, Section of Molecular Pharmacology and Pharmacogenetics, The Royal Hallamshire Hospital, Sheffield S10 2JF and* [2]*University of Sheffield, Department of Paediatrics, Section of Paediatric Haematology, The Children's Hospital, Sheffield, S10 2TH, UK.*

**Summary** As part of a programme assessing the pharmacokinetics of oral thiopurines given for lymphoblastic leukaemia, we assayed intracellular metabolites of mercaptopurine in children from all over the United Kingdom who were given a standard dose of the drug. The metabolites we measured, thioguanine nucleotides and methylmercaptopurines, are products of two competing metabolic pathways and would be expected to show an inverse correlation. A total of 327 children from 17 centres in the UK were studied. All were on the same therapeutic schedule of mercaptopurine. All had been on an unattenuated full protocol-directed dose (at least 75 mg $m^{-2}$) for a minimum of 7 days before assay. There was a very wide variation in the concentration of the two metabolites measured; the thioguanine nucleotides ranged from 0 to 1255 pmol per $8 \times 10^8$ red cells (median 289, lower quartile 210, upper quartile 377) and the methylmercaptopurine metabolites ranged from 0 to 46.3 nmol per $8 \times 10^8$ red cells (median 5.18, lower quartile 2.31, upper quartile 11.59). The anticipated negative correlation was not apparent, but the ratio between the two was not randomly distributed. No child had both metabolite concentrations in the upper quartiles, but in 32 (10%) children the concentration of both metabolites was in the lower quartile. Of the 32, only one metabolite was detected in four and none at all in six. The most likely explanation for these findings is that a minority of children with lymphoblastic leukaemia fail to take oral mercaptopurine either totally or intermittently. The extent of the problem is unknown, but we suspect it may be clinically important in at least 10% of patients.

**Keywords:** leukaemia; compliance; mercaptopurine

Cytotoxic thioguanine nucleotides formed from mercaptopurine are important in the continuing (maintenance) therapy of the common form of childhood lymphoblastic leukaemia (ALL). Patients who fail to form adequate amounts appear to be at higher risk of disease relapse (Lilleyman and Lennard, 1994). The reasons that individuals vary in their ability to produce these important intracellular compounds are not all clear, but one is inherited differences in activity of the enzyme thiopurine methyltransferase (TPMT). TPMT facilitates the formation of methylmercaptopurines at the expense of thioguanine nucleotides by a competing pathway. So very high thiopurine methyltransferase activity results in the formation of small amounts of thioguanine nucleotides and vice versa (Lennard *et al.*, 1990).

An alternative explanation for differences between patients is failure to comply with prescribed therapy. This possibility has received scant attention from paediatric onoclogists so far, but such data as there are all consistently suggest that the problem is clinically important. The topic has recently been reviewed by Davies and Lilleyman (1995).

We have now had the opportunity to assay intracellular metabolites on a large group of children from all over the United Kingdom and compare the concentration of methylmercaptopurines with thioguanine nucleotides. We thought this would allow us to distinguish between those with low thioguanine nucleotides due to high TPMT activity from those with low metabolites for other reasons – including poor compliance. This report describes our preliminary findings.

Correspondence: L Lennard
Received 3 January 1995; revised 25 April 1995; accepted 12 May 1995

### Materials and methods

### *Subjects and study design*

Clinicians with patients in the UK Medical Research Council UK ALL trials were invited to forward blood samples for measurement of mercaptopurine metabolites from children with all types of ALL receiving continuing chemotherapy. All were on trials UKALL X (1985–90) (Chessells *et al.*, 1995) or XI (1990 to date) (Lilleyman and Lennard, 1994).

Blood samples were requested at a time when the mercaptopurine dose was at a protocol-directed dose of at least 75 mg $m^{-2}$ for at least 7 preceding days, and not within 6 weeks of a red cell transfusion or within 8 weeks of a block of 'intensive' chemotherapy. A 5 ml aliquot was taken in a lithium heparin tube at routine venous access immediately before a monthly vincristine injection.

Continuing (maintenance) chemotherapy on all trials was similar and consisted of daily oral mercaptopurine and weekly oral methotrexate at standard doses of 75 mg $m^{-2}$ and 20 mg $m^{-2}$ respectively. The patients were generally instructed to take their tablets on an empty stomach at the same time each day. The children had full blood counts at least fortnightly to detect cytopenias. Protocol-directed drug dose reduction was made if neutropenia (neutrophils $<1 \times 10^9 l^{-1}$) or thrombocytopenia (platelets $<100 \times 10^9 l^{-1}$) occurred, and when the blood counts recovered above threshold a protocol-directed cycle of dose increments followed. All the children also received a monthly dose of intravenous vincristine and 5 days of oral prednisolone, irrespective of blood counts. A 5 day block of five-drug 'intensive' therapy given 3 months into continuing treatment was randomised in UKALL X but received by all in UKALL XI. In the latter study there was also a randomised 8 week 'third intensification' at weeks 35–43.

Red blood cell concentrations of thioguanine nucleotides and methylmercaptopurine metabolites were measured as previously described (Lennard and Singleton, 1992). Correlations were assessed for statistical significance by Spearman's rank correlation coefficient ($r_s$). Medians were compared by the Mann–Whitney *U*-test, and a two-tailed chi-squared test (with Yates' correction) was used to compare patient characteristics within subgroups of children. When more than one sample from any patient had been assayed, the values from the first sample were used for the purpose of the study.

### Results

Blood samples were forwarded from 17 UK centres over a 2.5 year period up to June 1994. They were received from 375 children (33 on UKALL X, 342 on UKALL XI, 138 girls and 237 boys, age 1–15 years, median 5 years). A total of 327 met the entry requirements for the study, of whom 115 were girls and 212 were boys (age 1–15 years, median 5 years). Of the 48 exclusions, seven samples could not be processed (lysed or clotted cells), four had no dose recorded and 34 were sampled at a lower than the standard dose (19–64 mg $m^{-2}$, median 50 mg $m^{-2}$). Three children were described as 'very sensitive' to mercaptopurine, i.e. unable to tolerate the standard dose. Two of these proved to have TPMT deficiency and have been reported elsewhere (Lennard *et al.*, 1993).

For the 327, red blood cell thioguanine nucleotides ranged from 0 to 1255 pmol per $8 \times 10^8$ red cells (median 289, lower quartile 210, upper quartile 377) and the methylmercaptopurine metabolites ranged from 0 to 46.3 nmol per $8 \times 10^8$ red cells (median 5.18, lower quartile 2.31, upper quartile 11.59). There was no significant difference between median metabolite concentrations in girls and boys.

For the group as a whole there was no statistically significant correlation between the thioguanine nucleotide concentrations and the methylmercaptopurine metabolite concentrations ($r_s = 0.007$, $P > 0.5$). A plot of the two metabolites is shown in Figure 1. From this it can be seen that the relationship between the two was not random and no child had both metabolite values in the upper quartiles. In contrast, in 32 children (10%) both metabolite values were within the lower quartiles, in four of whom only one metabolite was detectable and in a further six of whom no metabolites were detectable. If these 32 children were removed from the analysis there was a significant negative correlation between the concentration of thioguanine nucleotides and methylmercaptopurine metabolites ($r_s = -0.26$, $z = 4.46$, $P < 0.001$).

**Figure 1** The relationship between red blood cell (RBC) thioguanine nucleotides (6-TGNs) and methylmercaptopurine metabolites (MeMPs) for the 327 children studied. One datum point per child. Thioguanine nucleotides ranged from 0 to 1255 pmol per $8 \times 10^8$ red cells (median 289, lower quartile 210, upper quartile 377) and the methylmercaptopurine metabolites ranged from 0 to 46.3 nmol per $8 \times 10^8$ red cells (median 5.18, lower quartile 2.31, upper quartile 11.59).

Age and sex of the 32 'lower quartile' children did not differ significantly from the rest of the group. Ethnic origin is not recorded in the UKALL trial data, but it was apparent that 7 of the 32 had an obviously Asian family name compared with 9 of the 295 remaining children ($\chi^2 = 18.1$, $P < 0.001$). No data were available on the socioeconomic status of the 32 families.

### Discussion

In patients taking mercaptopurine there is an inverse relationship between TPMT activity and the intraerythrocyte concentration of thioguanine nucleotides (Lennard *et al.*, 1990). This is because the enzyme allows the formation of methylated metabolites via a competing pathway. It follows from this that there would be an expected inverse correlation between the concentrations of methylated metabolites and thioguanine nucleotides in patients at or near steady state after 7 days on a standardised dose. The fact that we have failed to demonstrate such a relationship in a large group of children appears to be because a proportion of them exhibited low concentrations of both types of metabolite, or, in some cases, no detectable metabolites at all. Although the reason is not known, the most likely explanation is that some of them failed to comply with their prescribed therapy either partially or completely. It is impossible to estimate the exact proportion, but it appears to be 10% or more of the children in the study.

We acknowledge that such a conclusion has to be tentative and that there are confounding influences in our patients of which we are ignorant. First, by collecting samples in the way we did we may have obtained results from a selected group of children. Some clinicians sent a sample from every child. Others sent samples on 'problem' children only, though these were mostly patients unable to tolerate normal doses of mercaptopurine and who had high metabolite concentrations. Secondly, mercaptopurine metabolism is obviously affected by factors other than TPMT or compliance, and no attempt was made in this study to assess absorption, for example. But we have yet to encounter a child in our local well-studied population in whom there are persistent low concentrations of both methylated mercaptopurines *and* thioguanine nucleotides; consequently, malabsorption is unlikely to be a common problem. Thus, despite these reservations, we feel we have collected more circumstantial evidence that some children with ALL fail to take their oral medication reliably.

While this may superficially be surprising in the face of a life-threatening disease, the problem is not unique to leukaemia and has also been recognised in children with epilepsy and diabetes (Shope, 1981), but reliably identifying non-compliance is very difficult. Some attempts have been made in children with cancer. We ourselves looked at a small group of local patients with ALL in whom we measured only the intracellular thioguanine nucleotides, but did so on more than one occasion when the patient had been on the same dose for at least 4 weeks. We took wide fluctuations as indicative of failure to comply. Of 22 children, four (18%) showed 2-fold or more variation and two subsequently admitted failure to take their tablets (Davies *et al.*, 1993). Other studies of children with malignant disease have used different techniques to reach broadly similar conclusions, suggesting non-compliance rates of 20–40% (Smith *et al.*, 1979; Tebbi *et al.*, 1986; Macdougall *et al.*, 1992), so our tentative figure of 10% in the present series may be an underestimate.

Why children might fail to comply is not clear. There are likely to be several influences. Despite the suggestion that adolescents and boys may be more delinquent, the age and sex distribution of our 32 'lower quartile' children was not clearly different from that in the rest. The only possibly important difference was that those of the 32 with Asian family names were strikingly in excess, which might reflect cultural differences and/or communication difficulties in different ethnic groups. It is also possible that socioeconomic status is relevant, but we have no data on that point.

We are not aware of any data on ethnic differences in TPMT activities among the Asian population of the Indian subcontinent, although ethnic differences have been documented for other racial groups. For example, American blacks have significantly lower TPMT activities than American white Caucasians, but the enzyme activity is similarly polymorphic in both populations (McLeod *et al.*, 1994). The possibility exists that the distribution of TPMT activities could differ in Asian populations, and this could contribute to the lower metabolite concentrations measured in children with Asian family names. In theory, a very high TPMT activity could remove most of the drug during intestinal and hepatic first-pass metabolism, and this could result in a low mercaptopurine availability for the red blood cell formation of both thioguanine nucleotides and methylmercaptopurine metabolites, as observed in some of our Asian children. This speculation is not supported by studies which have measured TPMT activities, thioguanine nucleotides and methylmercaptopurines in the same children. Those children with the highest red blood cell methylmercaptopurine metabolite concentrations had the highest red blood cell TPMT activities and vice versa (Lennard *et al.*, 1993).

Whatever the reason, poor compliance is potentially likely to put patients at higher risk of disease relapse. There is also another hazard. With the present policy of dose escalation in the face of normal blood counts there is a danger of severe myelosuppression if children suddenly start to take an inflated dose. For this reason we are increasingly inclined to the view that mercaptopurine metabolites should be monitored in all children on therapy for ALL, and those with constitutional high activity of TPMT identified at the outset. In this way potential non-compliers can be picked out at an early stage, and the mere act of surveillance may serve to reduce their number anyway.

In all analyses of clinical ALL trials to date full compliance and adequacy of oral antimetabolite drug effect has invariably (and naïvely) been assumed. If current cure rates for low-risk 'common' ALL are nudging 80% (Rivera *et al.*, 1993), it is tempting to wonder how much of the remaining 20% could be made up simply by ensuring that patients take all their tablets. The difficulty of this task should not be underestimated.

**Acknowledgements**
We are grateful to the following for sending samples on their patients: Dr CC Bailey, Dr L Ball, Dr V Broadbent, Prof JM Chessells, Dr S Dempsey, Dr P Darbyshire, Prof OB Eden, Dr BES Gibson, Dr I Hann, Dr E Hill, Dr FGH Hill, Dr J Kernahan, Dr J Kingston, Dr I Lewis, Dr D Moir, Dr PH Morris Jones, Dr T Nicole, Dr S Meller, Dr A Oakhill, Dr AM O'Hea, Dr M Plazcek, Dr DH Pamphilion, Dr M Radford, Dr GP Summerfield, Dr D Stevens, Dr RF Stevens, Dr EN Thompson, Dr DA Walker, Dr DKW Webb, Dr A Will. LL and JW were supported by the Leukaemia Research Fund.

### References

CHESSELLS JM, BAILEY CC AND RICHARDS SM. (1995). Intensification of treatment improves survival for all children with lymphoblastic leukaemia: results of MRC UKALL X. *Lancet*, **345**, 143–148.

DAVIES HA AND LILLEYMAN JS. (1995). Compliance with oral chemotherapy in childhood lymphoblastic leukaemia. *Cancer Treat. Rev.*, **21**, 93–103.

DAVIES HA, LENNARD L AND LILLEYMAN JS. (1993). Variable mercaptopurine metabolism in children with leukaemia: a problem of non-compliance? *B. Med. J.*, **306**, 1239–1240.

LENNARD L AND SINGLETON H. (1992). High performance liquid chromatographic assay of the methyl and nucleotide metabolites of 6-mercaptopurine; quantitation of red blood cell 6-thiogunanine nucleotide, 6-thioinosinic acid and 6-methylmercaptopurine metabolites in a single sample. *J. Chromatogr.*, **583**, 83–90.

LENNARD L, LILLEYMAN JS, VAN LOON JA AND WEINSHILBOUM RM. (1990). Genetic variation in response to 6-mercaptopurine for childhood acute lymphoblastic leukaemia. *Lancet*, **336**, 225–229.

LENNARD L, GIBSON BES, NICOLE T AND LILLEYMAN JS. (1993). Congenital thiopurine methyltransferase deficiency and 6-mercaptopurine toxicity during treatment for acute lymphoblastic leukaemia. *Arch. Dis. Child.*, **69**, 577–579.

LILLEYMAN JS AND LENNARD L. (1994). Mercaptopurine metabolism and risk of relapse in childhood lymphoblastic leukaemia. *Lancet*, **343**, 1188–1190.

MACDOUGAL LG, MCELLIGOTT SE, ROSS E, GREEF MC AND POOLE JE. (1992). Pattern of 6-mercaptopurine urinary excretion in children with acute lymphoblastic leukaemia: urinary assays as a measure of drug compliance. *Ther. Drug. Monit.*, **14**, 371–375.

MCLEOD HL, LIN J-S, SCOTT EP, PUI C-H AND EVANS WE. (1994). Thiopurine methyltransferase activity in American white subjects and black subjects. *Clin. Pharmacol. Ther.*, **55**, 15–20.

RIVERA GK, PINKEL D, SIMONE JV, HANCOCK ML AND CHRIST WM. (1993). Treatment of acute lymphoblastic leukaemia; 30 years' experience at St. Jude Children's Research Hospital. *N. Engl. J. Med.*, **329**, 1289–1295.

SHOPE JS. (1981). Medication compliance. *Pediatr. Clin. N. Am.*, **28**, 5–21.

SMITH SD, ROSEN D, TRUEWORTHY RC AND LOWMAN JT. (1979). A reliable method for evaluation of drug compliance in children with cancer. *Cancer*, **43**, 169–173.

TEBBI CK, CUMMINGS KM, ZEVON MA, SMITH L, RICHARDS M AND MALLON J. (1986). Compliance of pediatric and adolescent cancer patients. *Cancer*, **58**, 1179–1184.